# Self-Administration of Ethanol, Cocaine, or Nicotine Does Not Decrease the Soma Size of Ventral Tegmental Area Dopamine Neurons

**DOI:** 10.1371/journal.pone.0095962

**Published:** 2014-04-22

**Authors:** Michelle S. Mazei-Robison, Raghu Appasani, Scott Edwards, Sunmee Wee, Seth R. Taylor, Marina R. Picciotto, George F. Koob, Eric J. Nestler

**Affiliations:** 1 Fishberg Department of Neuroscience and Friedman Brain Institute, Icahn School of Medicine at Mount Sinai, New York, New York, United States of America; 2 Department of Physiology and Neuroscience Program, Michigan State University, East Lansing, Michigan, United States of America; 3 Committee on the Neurobiology of Addictive Disorders, Scripps Research Institute, La Jolla, California, United States of America; 4 Department of Physiology, Louisiana State University School of Medicine, New Orleans, Louisiana, United States of America; 5 Department of Molecular Therapeutics, Florida Campus, Scripps Research Institute, Jupiter, Florida, United States of America; 6 Department of Psychiatry, Yale University School of Medicine, New Haven, Connecticut, United States of America; University of California San Diego, United States of America

## Abstract

Our previous observations show that chronic opiate administration, including self-administration, decrease the soma size of dopamine (DA) neurons in the ventral tegmental area (VTA) of rodents and humans, a morphological change correlated with increased firing rate and reward tolerance. Given that a general hallmark of drugs of abuse is to increase activity of the mesolimbic DA circuit, we sought to determine whether additional drug classes produced a similar morphological change. Sections containing VTA were obtained from rats that self-administered cocaine or ethanol and from mice that consumed nicotine. In contrast to opiates, we found no change in VTA DA soma size induced by any of these other drugs. These data suggest that VTA morphological changes are induced in a drug-specific manner and reinforce recent findings that some changes in mesolimbic signaling and neuroplasticity are drug-class dependent.

## Introduction

Despite the importance of ventral tegmental area (VTA) dopamine (DA) neurons in drug-dependent behaviors, little is known about the structural plasticity induced in these cells by drugs of abuse. Chronic opiate administration, including self-administration, decreases the soma size of VTA DA neurons in rodents and humans [1], [2], [3], [4]. This decrease is dependent on AKT signaling and increased neuronal activity, and mediates reward tolerance [2], [3]. In spite of the increase in VTA DA firing rate, DA output to the nucleus accumbens (NAc) is decreased, suggesting that the structural changes alter mesolimbic DA circuit function, contributing to the change in reward behavior [3]. The question remains whether other classes of abused drugs affect the structure of VTA DA neurons, as all abused drugs increase the function of the mesolimbic DA circuit. Given recent evidence that modulation of neurotrophic signaling within this circuit differentially affects cocaine and morphine reward [5], [6], we sought to determine whether chronic administration of cocaine, ethanol, or nicotine decreases VTA DA soma size. In contrast to chronic opiate administration, chronic administration of these drugs did not alter VTA DA soma size in rodents, highlighting the importance of investigating class-specific neuroadaptations induced by drugs in addition to their common actions.

## Materials and Methods

### Animals

For ethanol and cocaine self-administration studies, male Wistar rats (Charles River Laboratories) were housed in a temperature-controlled vivarium in groups of 2–3 with food and water available *ad libitum*. For nicotine studies, male c57Bl/6J mice (Jackson Laboratories) were group-housed (4–5/cage) with food and water available *ad libitum* in a temperature-controlled vivarium on a 12 hour light-dark cycle. All animal protocols were approved by Institutional Animal Care and Use Committees [Yale University (mice) or the Scripps Research Institute (rats)] and complied with strict guidelines set in the Guide for the Care and Use of Laboratory Animals of the National Institutes of Health.

### Ethanol Self-Administration

Ethanol self-administration was completed in male Wistar rats as described previously [7]. Briefly, following ten sessions of ethanol (10% w/v) self-administration, rats were split into two groups, matched for self-administration. The “ethanol-dependent” group was exposed to chronic, intermittent ethanol vapors (14 h on vapor and 10 h off) for ≥12 weeks (n = 6). Rats in the “ethanol-exposed” non-dependent group were exposed to air alone (n = 6). Ethanol-naïve rats (n = 6) received neither ethanol self-administration nor vapor exposure but were handled regularly. Rats were sacrificed 6–8 hours after ethanol-vapor exposure via perfusion with 4% paraformaldehyde.

### Cocaine Self-Administration

For the cocaine studies, male Wistar rats were implanted with a silastic catheter into the right external jugular vein, allowed to recover, then trained to self-administer cocaine (0.5 mg/kg/infusion) in operant conditioning chambers as described previously [8]. Following stable acquisition, rats were divided into two groups, balanced by cocaine intake. The short-access (ShA) group (n = 3) was allowed to self-administer cocaine for 1 hour, while the long-access (LgA) group (n = 3) was allowed 6 hours of self-administration for 10 days. Cocaine-naïve rats (n = 3) received neither cocaine exposure nor intravenous catheterization. 24 hours after the last cocaine administration rats were perfused with 4% paraformaldehyde.

### Nicotine Drinking

For nicotine experiments, c57Bl/6J mice were used as described [9]. Mice were given 2% saccharin (n = 5) or 200 μg/mL nicotine in 2% saccharin in the drinking water (n = 6) for 21 days and were then immediately perfused with 4% paraformaldehyde.

### Immunohistochemistry and Confocal Microscopy

All brains were post-fixed in 4% paraformaldehyde and transferred to 30% sucrose-PBS. 30 μm sections containing VTA were processed as described [3]. Briefly, sections were incubated with a monoclonal antibody to tyrosine hydroxylase (Sigma) and a fluorescent secondary antibody (Jackson ImmunoResearch) was used for detection. Sections were scanned using a Zeiss LSM 710 microscope and 3D reconstruction and size analysis were completed using Volocity software (PerkinElmer). A blind analysis of soma size was completed by two investigators for each drug treatment. One-way ANOVA was used to test for soma size differences in cocaine and ethanol experiments and a Student's unpaired t-test was used for the nicotine study.

## Results

**Figure 1:**
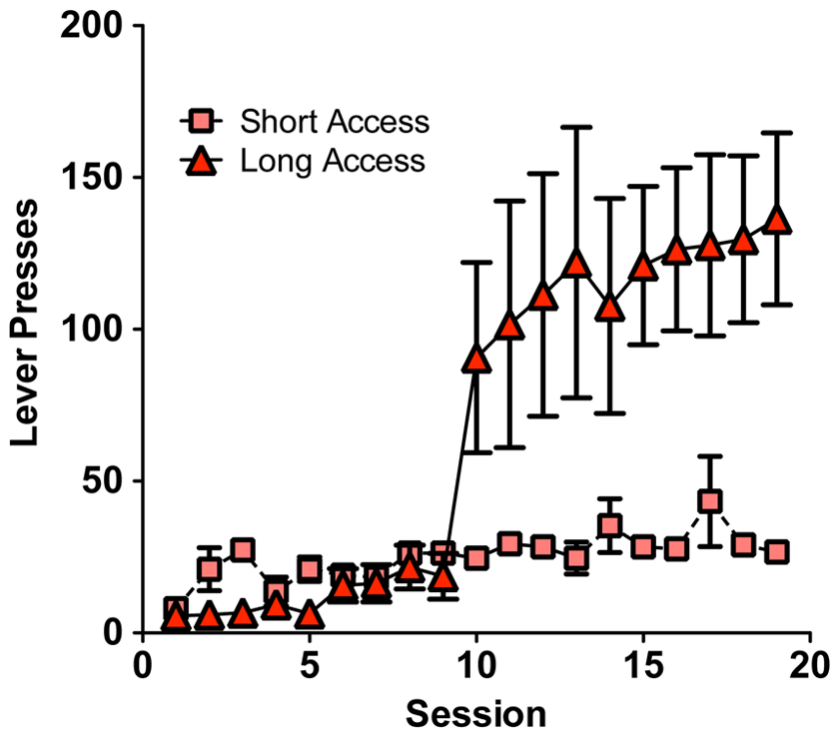
Rats given extended access to cocaine (triangles) escalate cocaine self-administration compared to rats allowed short access (squares). Rats were trained to self-administer 0.5 mg/kg/infusion of cocaine in one-hour sessions under a fixed-ratio 1 schedule for nine days. After nine baseline sessions, the rats were divided into two groups balanced by cocaine intake on the last two sessions. One group of rats was allowed to self-administer cocaine in one-hour sessions (short access, ShA) while the other group of rats self-administered cocaine in six-hour sessions (long access, LgA) for ten additional days (escalation sessions). n = 3 rats/group.

Drug intake data for animals used in the study are detailed in Tables 1, 2, and 3. As shown previously, ethanol-dependent rats achieve significant blood alcohol levels, averaging a maximum 171±10.1 mg/dl at the end of the exposure period. Rats given extended access to cocaine show increased self-administration compared to short access, and higher total intake (Table 1, Figure 1). Home-cage intake of a nicotine-saccharin solution was similar to previous studies (Table 2 and 3), that yield significant serum nicotine/cotinine levels [9].

**Table 1:** Cocaine intake data for rats allowed to self-administer cocaine during 1 hr (short access) or 6 hr (long access) sessions.

Rat#	Group	Total cocaine intake (mg/kg)	Last session intake (mg/kg)
1	Naïve	0	0
2	Naïve	0	0
3	Naïve	0	0
408	Short access	236	14.0
411	Short access	231	13.5
420	Short access	251	12.5
412	Long access	318	43.0
419	Long access	695	69.5
430	Long access	909	92.0

**Table 2:** Home-cage intake of saccharin and nicotine drinking water (ml water/day/mouse).

Group	Week 1	Week 2	Week 3
Saccharin Group 1	7.23	7.27	7.25
Saccharin Group 2	4.63	4.20	4.56
Nicotine Group 1	3.54	3.60	3.95
Nicotine Group 2	4.06	3.43	4.22

**Table 3:** Average amount of nicotine consumed (mg/kg/day).

Group	Week 1	Week 2	Week 3
Saccharin Group 1	0	0	0
Saccharin Group 2	0	0	0
Nicotine Group 1	28.4	28.8	31.6
Nicotine Group 2	32.5	27.4	33.7

**Figure 2:**
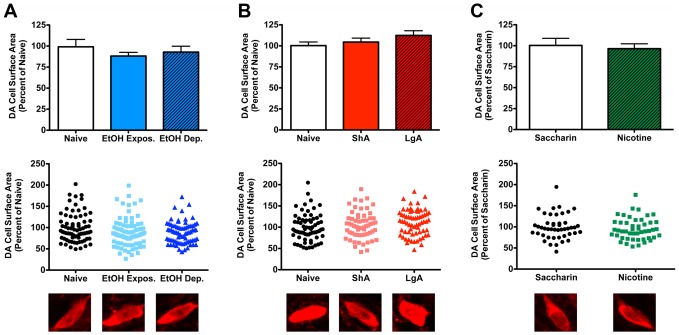
VTA DA soma size is not altered by self-administration of ethanol (A), cocaine (B), or nicotine (C). Average soma size is shown in top graph, individual cell data are presented in the middle graph, and representative cell images are shown below. (A) Ethanol-exposed or -dependent rats exhibited no difference in VTA soma size compared to naïve animals. n = 6 rats/group, 4–23 cells/rat. (B) Rats with either short (ShA) or long (LgA) access to cocaine exhibited no difference in VTA soma size compared to naïve animals. n = 3 rats/group, 19–28 cells/rat. (C) Mice that consumed nicotine for 21 days exhibited no significant decrease in VTA DA soma size from saccharin drinking controls. n = 5 (saccharin) or 6 (nicotine) mice, 5–13 cells/mouse.

Following chronic drug administration, the soma size of VTA DA neurons was examined using immunohistochemistry. As shown in Figure 2, there were no differences for any of the 3 different drug treatments. There was no statistical difference between naïve, ethanol-exposed, and ethanol-dependent rats (Fig. 2A; one-way ANOVA, F (2,15) = 0.6339, p = 0.5441). Although there was a trend for an increase in soma size in the long-access cocaine rats, there was no statistical difference between the naïve, short-access, and long-access groups (Fig. 2B; one-way ANOVA, F (2,6) = 1.565, p = 0.2839). Similarly, there was no difference in VTA DA soma size between saccharin and nicotine-drinking mice (Fig. 2C; unpaired Student's t-test, t(9) = 0.3876, p = 0.7073).

## Discussion

We have shown previously that chronic administration of opiates, including morphine and heroin, decreases the soma size of VTA DA neurons, which contributes to drug-induced behavioral changes, specifically reward tolerance [2]. The current report shows that this morphological change is unique to opiates, and may offer key insights into specific interventions for opiate addiction by identifying the underlying signaling mechanisms in the VTA. Notably, we have shown previously that decreased AKT activity is critical for opiate-induced changes in morphology and behavior [2], [3] and cocaine administration does not appear to alter VTA AKT activity (MSM and EJN unpublished observations). One consequence of decreasing AKT activity is alteration of the activity of AKT's substrates, such as glycogen synthase 3 beta (GSK3β), which is normally phosphorylated by AKT and leads to a decrease in GSK3β activity. Alteration of GSK3β activity has been shown to alter neuronal size and structure [10], and while we found that changes in GSK3β activity were not required for our opiate-induced changes [3], a recent study conducted in a mouse model of mania, the *ClockΔ19* mice, suggests that increased GSK3β activity may mediate a decrease in VTA DA soma size and increased VTA DA activity similar to that which we observe with chronic opiate administration [11]. Specifically, Coque et al. [11] found that *ClockΔ19* mice exhibit decreased VTA DA soma size and increased VTA DA activity and that these differences can be normalized by lithium treatment, a known GSK3β inhibitor. This study additionally serves to highlight the functional relevance of the VTA DA soma size change as rescue of the soma size decrease by lithium or overexpression of an inwardly rectifying potassium channel also normalized locomotor- and anxiety-related behaviors [11], [12].

One caveat to the current data and their interpretation is that we only examined soma size changes induced by chronic drug administration; we did not examine the effect of drug withdrawal. Withdrawal from both opiates and cannabinoids has been shown to decrease VTA DA soma size [13], [14], so this remains a possibility for cocaine, nicotine, and ethanol. While the ethanol-dependent rats in this study could be considered to be in acute withdrawal given their low BAL at sacrifice, this is the normal withdrawal they go through everyday during the 10 hour daily absence of EtOH vapor, and not a prolonged withdrawal or abstinence time-point. In this context, opiates appear unique, as they induce changes in soma size both with chronic use [2], [3], [4] and withdrawal [4], [13], whereas cannabinoids only induce a decrease in soma size during withdrawal [14].

Changes in dendritic spine number or complexity are another form of structural plasticity that is differentially affected by drugs of abuse. For example, chronic opiate and stimulant drug administration has been shown to have opposite effects on dendritic spine plasticity in the reward circuit (reviewed in [15]). Specifically, morphine decreases dendritic spine number and complexity in the NAc and prefrontal cortex (PFC) [16], while stimulants such as cocaine and amphetamine induce increases in both brain regions [17]. Similar to opiates, cannabinoid withdrawal decreases dendritic spine density in the NAc shell [14]. Whether chronic exposure to these various drugs similarly alters dendritic spines in the VTA is an open question. It has been reported that a single acute injection of cocaine increases dendritic spine density in a subset of VTA neurons [18], and dendritic length is increased in adolescent rats treated repeatedly with amphetamine [19], results consistent with chronic stimulant effects in the NAc and PFC. While an increase in spine number would generally be thought to contribute to increased mesolimbic circuit function and increased behavioral responses to drug exposure, the fact that both increased and decreased spine complexity are associated with locomotor sensitization [15] underscores the challenge in equating structural changes in a specific brain region to circuit function and behavioral output.

Our data presented here are in line with dendritic spine data in NAc and PFC and support the notion that drugs of abuse can induce distinct neuroadaptations, even within the commonly targeted mesolimbic DA circuit. Identifying these differences, in addition to the similarities, will be critical in both understanding the underlying etiology of drug addiction and in proposing novel therapeutic interventions.

## References

[1] Sklair-Tavron L, Shi WX, Lane SB, Harris HW, Bunney BS, et al (1996) Chronic morphine induces visible changes in the morphology of mesolimbic dopamine neurons. Proc Natl Acad Sci U S A 93: 11202–11207. 8855333 10.1073/pnas.93.20.11202 PMC38308

[2] Russo SJ, Bolanos CA, Theobald DE, DeCarolis NA, Renthal W, et al (2007) IRS2-Akt pathway in midbrain dopamine neurons regulates behavioral and cellular responses to opiates. Nat Neurosci 10: 93–99. 17143271 10.1038/nn1812

[3] Mazei-Robison MS, Koo JW, Friedman AK, Lansink CS, Robison AJ, et al (2011) Role for mTOR signaling and neuronal activity in morphine-induced adaptations in ventral tegmental area dopamine neurons. Neuron 72: 977–990. 22196333 10.1016/j.neuron.2011.10.012 PMC3246191

[4] Chu NN, Zuo YF, Meng L, Lee DY, Han JS, et al (2007) Peripheral electrical stimulation reversed the cell size reduction and increased BDNF level in the ventral tegmental area in chronic morphine-treated rats. Brain Res 1182C: 90–98. 10.1016/j.brainres.2007.08.086 PMC2703674 17945205

[5] Koo JW, Mazei-Robison MS, Chaudhury D, Juarez B, LaPlant Q, et al (2012) BDNF is a negative modulator of morphine action. Science 338: 124–128. 23042896 10.1126/science.1222265 PMC3547365

[6] Graham DL, Krishnan V, Larson EB, Graham A, Edwards S, et al (2009) Tropomyosin-related kinase B in the mesolimbic dopamine system: region-specific effects on cocaine reward. Biol Psychiatry 65: 696–701. 18990365 10.1016/j.biopsych.2008.09.032 PMC2738869

[7] Edwards S, Guerrero M, Ghoneim OM, Roberts E, Koob GF (2012) Evidence that vasopressin V1b receptors mediate the transition to excessive drinking in ethanol-dependent rats. Addict Biol 17: 76–85. 21309953 10.1111/j.1369-1600.2010.00291.x PMC3178679

[8] Wee S, Vendruscolo LF, Misra KK, Schlosburg JE, Koob GF (2012) A combination of buprenorphine and naltrexone blocks compulsive cocaine intake in rodents without producing dependence. Sci Transl Med 4: 146ra110. 10.1126/scitranslmed.3003948 PMC3448552 22875830

[9] King SL, Caldarone BJ, Picciotto MR (2004) Beta2-subunit-containing nicotinic acetylcholine receptors are critical for dopamine-dependent locomotor activation following repeated nicotine administration. Neuropharmacology 47 Suppl 1132–139. 15464132 10.1016/j.neuropharm.2004.06.024

[10] van Diepen MT, Parsons M, Downes CP, Leslie NR, Hindges R, et al (2009) MyosinV controls PTEN function and neuronal cell size. Nat Cell Biol 11: 1191–1196. 19767745 10.1038/ncb1961 PMC2756284

[11] Coque L, Mukherjee S, Cao JL, Spencer S, Marvin M, et al (2011) Specific Role of VTA Dopamine Neuronal Firing Rates and Morphology in the Reversal of Anxiety-Related, but not Depression-Related Behavior in the ClockDelta19 Mouse Model of Mania. Neuropsychopharmacology 36: 1478–1488. 21430648 10.1038/npp.2011.33 PMC3096816

[12] Roybal K, Theobold D, Graham A, DiNieri JA, Russo SJ, et al (2007) Mania-like behavior induced by disruption of CLOCK. Proc Natl Acad Sci U S A 104: 6406–6411. 17379666 10.1073/pnas.0609625104 PMC1851061

[13] Spiga S, Serra GP, Puddu MC, Foddai M, Diana M (2003) Morphine withdrawal-induced abnormalities in the VTA: confocal laser scanning microscopy. Eur J Neurosci 17: 605–612. 12581178 10.1046/j.1460-9568.2003.02435.x

[14] Spiga S, Lintas A, Migliore M, Diana M (2010) Altered architecture and functional consequences of the mesolimbic dopamine system in cannabis dependence. Addict Biol 15: 266–276. 20477755 10.1111/j.1369-1600.2010.00218.x

[15] Russo SJ, Mazei-Robison MS, Ables JL, Nestler EJ (2009) Neurotrophic factors and structural plasticity in addiction. Neuropharmacology 56 Suppl 173–82. 10.1016/j.neuropharm.2008.06.059 PMC2635335 18647613

[16] Robinson TE, Kolb B (1999) Morphine alters the structure of neurons in the nucleus accumbens and neocortex of rats. Synapse 33: 160–162. 10400894 10.1002/(SICI)1098-2396(199908)33:2<160::AID-SYN6>3.0.CO;2-S

[17] Robinson TE, Kolb B (1999) Alterations in the morphology of dendrites and dendritic spines in the nucleus accumbens and prefrontal cortex following repeated treatment with amphetamine or cocaine. Eur J Neurosci 11: 1598–1604. 10215912 10.1046/j.1460-9568.1999.00576.x

[18] Sarti F, Borgland SL, Kharazia VN, Bonci A (2007) Acute cocaine exposure alters spine density and long-term potentiation in the ventral tegmental area. Eur J Neurosci 26: 749–756. 17686047 10.1111/j.1460-9568.2007.05689.x

[19] Mueller D, Chapman CA, Stewart J (2006) Amphetamine induces dendritic growth in ventral tegmental area dopaminergic neurons in vivo via basic fibroblast growth factor. Neuroscience 137: 727–735. 16338078 10.1016/j.neuroscience.2005.09.038

